# Cardiac telerehabilitation: current status and future perspectives

**DOI:** 10.1007/s12471-023-01833-9

**Published:** 2023-12-12

**Authors:** Rutger W. M. Brouwers, Martijn Scherrenberg, Hareld M. C. Kemps, Paul Dendale, Johan A. Snoek

**Affiliations:** 1 Department of Cardiology, Máxima Medical Center, Veldhoven, The Netherlands; 2 Heart Centre, Catharina Hospital, Eindhoven, The Netherlands; 3 Department of Industrial Design, Eindhoven University of Technology, Eindhoven, The Netherlands; 4 Heart Centre Hasselt, Jessa Hospital, Hasselt, Belgium; 5 Faculty of Medicine and Health Sciences, Antwerp University, Antwerp, Belgium; 6 Faculty of Medicine and Life Sciences, Hasselt University, Diepenbeek, Belgium; 7 Isala Heart Centre, Zwolle, The Netherlands; 8 Sports Medicine Department, Isala, Zwolle, The Netherlands

**Keywords:** Cardiac rehabilitation, Cardiac telerehabilitation, Digital health, Coronary artery disease, Chronic heart failure, Secondary prevention

## Abstract

Multidisciplinary cardiac rehabilitation (CR) improves the prognosis and quality of life of patients with cardiovascular disease and has therefore received strong recommendations in international guidelines for the treatment of patients with chronic coronary syndromes and chronic heart failure. Aiming to both resolve several barriers that impede participation in CR and to improve the effectiveness of CR, cardiac telerehabilitation (CTR) has emerged as a cost-effective alternative to traditional, centre-based CR. Although the body of evidence for the feasibility and effectiveness of CTR is large and still growing, real-life implementations are scarce, which may be due to insufficient knowledge about CTR interventions and due to the challenges its implementation comes with. Up to now, mainly exercise-related core components of CR and e‑coaching have been investigated in the setting of CTR. Translation of research findings to clinical practice may be hampered by methodological limitations present in most CTR studies, being selection bias of participants, lack of long-term follow-up, heterogeneity of studied interventions and the lack of robust outcome measures. Besides conducting highly needed implementation studies for CTR interventions, their implementation could be facilitated by the development of guideline-based, multidisciplinary and personalised CTR programmes and widespread reimbursement for CTR.

## Introduction

Participation in multidisciplinary cardiac rehabilitation (CR) improves the prognosis and quality of life of patients with cardiovascular disease and has received class IA recommendations in international guidelines for the treatment of patients with chronic coronary syndromes and chronic heart failure (CHF) [1, 2]. Moreover, participation in CR is cost-effective compared with non-participation [3] and increased utilisation of CR results in societal cost-savings [4]. Over the past decades, CR has evolved from a one-dimensional exercise-based intervention to a multidimensional intervention that includes risk factor modification, education and treatment by psychologists, dieticians and/or social workers. For patients with coronary artery disease (CAD), participation in CR reduces all-cause mortality (by 32–35% [5–7]) and cardiovascular mortality (by 26% [8]), major adverse cardiac events and all-cause hospitalisations (by 23% [8]), and improves quality of life (QoL) [8]. For patients with CHF (mostly heart failure with reduced ejection fraction (HFrEF)), the use of CR results in significant reductions (of 20–30%) in all-cause and heart failure related hospitalisations and clinically relevant improvements in QoL [9–11]. For patients with atrial fibrillation or those having undergone valve surgery, evidence to support benefits of CR is less abundant [12, 13], although cohort studies have demonstrated improvements in mortality, rehospitalisation and health-related QoL [14–16]. Despite the aforementioned clinical and economic benefits, less than half of the eligible patients complete a CR programme [17]. The cause of low participation and completion rates is multifactorial, involving barriers at patient level (e.g., comorbidities, distance to nearest CR provider), healthcare professional level (e.g., lack of awareness of the benefits of CR) and healthcare system level (e.g., limited training facilities, lack of reimbursement of CR) [10, 18].

**Fig. 1:**
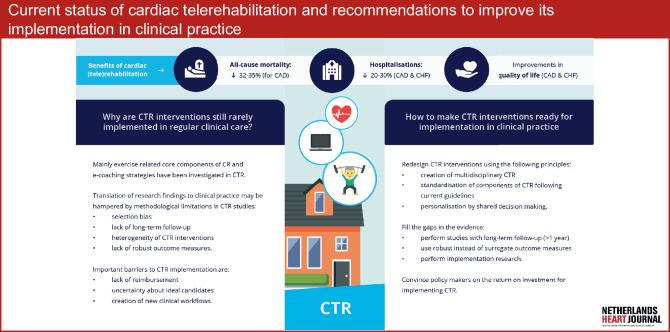
Infographic

Cardiac telerehabilitation (CTR) is an alternative to centre-based CR in which one or more treatment modules of the CR programme are delivered in a patient’s home environment, using wearable devices and remote communication between healthcare professionals and patients. Implementation of CTR may increase overall CR utilisation and completion by resolving one or more of the aforementioned barriers. At a patient and professional level, for example, CTR may better suit the needs and preferences of a significant proportion of eligible patients, likely resulting in increased referral rates and improvement in programme enrolment and completion. At a system level, the delivery of CTR requires less personnel and training facilities per patient than centre-based CR. Therefore, the implementation of CTR facilitates increasing the number of CR participants without substantial expansion of these increasingly scarce resources, besides inevitable initial investments in technology and training of personnel. Multiple systematic reviews and meta-analyses have demonstrated that CTR is a cost-effective alternative to centre-based CR for patients with CAD and CHF, resulting in similar clinical and economic benefits [19–23]. Nevertheless, CTR interventions are often limited to research settings and are still rarely implemented in regular care, which may at least partly be due to insufficient knowledge of the possibilities of CTR and of the challenges its implementation comes with. In this narrative review, we aim to provide an overview of which core components of CR can be delivered using CTR, address methodological limitations of CTR studies, and discuss barriers to CTR implementation in clinical practice (Fig. 1).

## Cardiac telerehabilitation for core components of CR

Guidelines recommend prescribing a CR programme that is personalised to the individual patient’s needs, taking into account disease and patient characteristics and personal rehabilitation goals [24]. Core components of a comprehensive multidisciplinary CR programme should include patient assessment, physical activity (PA) counselling and exercise training, diet and nutritional counselling, risk factor control, patient education, psychosocial management and vocational advice [25]. Below we will provide an overview of the scientific evidence for the remote delivery of the exercise and non-exercise related CR core components respectively.

### Evidence for the remote delivery of exercise and non-exercise related CR core components: **key messages**

- To optimise exercise capacity, increasing total energy expenditure with moderate-intensity continuous training is currently preferred for CTR
- CTR may be more effective than centre-based CR in sustaining physical activity and exercise capacity when behavioural change techniques are combined with prolonged periods of self-monitoring
- Remotely supervised digital health interventions for cardiovascular risk factor control have been mainly investigated outside the CR setting, but can be incorporated in CTR interventions
- Other non-exercise related core components (such as nutritional or psychosocial counselling) can be effectively delivered using CTR

### Patient assessment and monitoring

Whereas some components of patient assessment and monitoring throughout the CTR programme still require face-to-face contact (e.g., blood testing, exercise testing), many components can be executed outside the CR centre using digital health solutions, questionnaires and remote communication, as also summarised previously [26]. For example, vital parameters can be measured by the patient using wearable sensors; psychosocial status and even physical fitness can be assessed using validated questionnaires [26]. To evaluate whether a patient is eligible to participate in CTR, digital readiness can be assessed using a recently validated questionnaire [27].

### Exercise-related core components: PA counselling and exercise training

The Dutch multidisciplinary guideline for CR distinguishes 19 specific goals without specifying practical recommendations for exercise therapy. An expert panel recently simplified exercise prescription in CR by describing five exercise-related clusters out of these 19 goals: 1) reducing exercise-related anxiety; 2) exploring physical limits and coping with physical limitations; 3) optimal work resumption; 4) optimising exercise capacity; 5) developing and maintaining a physically active lifestyle and optimising cardiovascular risk factors [28].

The goals *optimising exercise capacity* and *work resumption *only differ with respect to work-specific resistance training and can easily be merged for exercise prescription. To increase exercise capacity in patients with CAD and CHF, both exercise intensity and a patient’s total energy expenditure should be optimised [29]. Although high-intensity interval training (HIIT) protocols might have added benefits over moderate-intensity continuous training (MICT) [30], it is still uncertain which training modality is best suited for the achievement of this goal [29]. A small number of randomised controlled trials have shown the effectiveness and safety of HIIT in a home-based setting in patients with CAD [31, 32]. However, the CTR addendum to the Dutch multidisciplinary CR guideline solely recommends continuous training, as evidence to recommend home-based HIIT is currently insufficient [33]. Hence, increasing total energy expenditure with MICT is currently preferred when executing CTR in a home-based setting. Subsequently, the preferred exercise modality, frequency, intensity and duration should be selected based on shared decision-making with the patient [29, 34].

Both the goals *reducing exercise-related anxiety* and *exploring physical limits and coping with physical limitations* relate to a physical limit. By gradually increasing exercise intensity during supervised aerobic and resistance training, patients learn to either cross or respect their limits [28]. Likewise, exercise-based games can be used to reduce exercise anxiety and explore physical limits. Although home-based aerobic training is recommended after a period of supervised training [28], these training characteristics and the lack of direct supervision might be less ideal for certain patients, but scientific evidence in this regard is lacking.

The objective of the goal *developing and maintaining a physically active lifestyle and optimising cardiovascular risk factors *is to change exercise behaviour in the long term. It is well-known that adherence to PA guidelines declines after completion of centre-based CR [35]. Long-term and home-based programmes that gradually increase exercise volume may be effective in supporting long-lasting lifestyle changes [35], although the optimal form and duration of CR are still unknown [36]. With CTR, the programme length can be extended beyond the traditional CR duration of 3 months. In several recent trials, a relapse in PA could not be prevented by the use of CTR [37–39]. In the long-term follow-up of the FIT@Home trial in patients with CAD and low residual cardiovascular risk, objectively measured PA declined to baseline levels after 4 years in both centre-based CR and CTR. However, when applying a stronger focus on behavioural change combined with prolonged periods of monitoring, CTR may in fact be more effective than centre-based CR in sustaining PA and exercise capacity [38, 40, 41]. The Telerehab III trial demonstrated that centre-based CR plus CTR (total duration of 9 months) led to better peak VO2_peak_ (22 ± 6 vs. 20 ± 6 ml/kg/min) and subjectively assessed PA levels after 2 years as compared with 12 weeks of centre-based CR alone [38].

### Non-exercise related core components

Evidence for the use of CTR for other, non-exercise related core components of CR (risk factor control, diet and nutritional counselling, patient education, psychosocial management and vocational advice) is relatively scarce. Moreover, remotely supervised digital health interventions for cardiovascular risk factor control have been mainly investigated outside the CR setting (e.g., in a primary prevention setting). Considerable overlap, however, exists between CTR interventions and such risk factor targeting interventions. Therefore, we believe that successful risk factor targeting interventions could easily be incorporated into a CR or CTR setting.

With respect to cardiovascular risk factor control, a systematic review of web-based interventions in middle-aged and older people with one or more risk factors or established cardiovascular disease found modest improvements in patients’ cardiovascular risk profiles: for example, systolic blood pressure −2.66 mm Hg (95% CI −3.81 to −1.52); change in weight −1.34 kg (95% CI −1.91 to −0.77); LDL-cholesterol −2.18 mg/dl (95% CI −3.96 to −0.41) [42]. Effects were more pronounced in studies with short-term (< 12 months) follow-up when compared with those with longer follow-up, and in studies that tested blended interventions (online applications combined with human support). No evidence was found, however, for an effect on incident cardiovascular disease (e.g., myocardial infarction, heart failure, stroke and peripheral arterial disease). Similarly, remotely supervised interventions for smoking cessation have also shown to be effective [26].

Regarding nutritional counselling, dietary intervention studies performed within a CR setting have found positive effects of e‑coaching strategies on changes in nutrition knowledge by using a simple messaging application, and better adherence to dietary recommendations with semi-personalised advice, motivational reminders and digital support with text messaging [43, 44]. Likewise, virtual education in CR can improve patients’ understanding of their disease, improve self-confidence and self-management and facilitate behavioural change [45, 46]. E‑coaching may have positive effects on psychosocial health in both medium (3–6 months) and long-term interventions (> 6 months), as demonstrated by a systematic review analysing 19 studies performed in a CR setting [19]. Although the studied interventions were highly heterogeneous, these results imply that psychosocial support by e‑coaching could be an effective alternative for centre-based psychosocial counselling.

## Methodological limitations of CTR studies

Although the effectiveness of CTR as an alternative to centre-based CR has been extensively demonstrated, a number of methodological limitations of CTR studies may hamper the translation of research findings to clinical practice. The most important limitations are selection bias of study participants, lack of long-term follow-up, heterogeneity of the study population and type and duration of CTR interventions, and lack of robust outcome measures.

### Methodological limitations of CTR studies: **key messages**

The most important methodological limitations of CTR studies include:

- Selection bias of study participants
- Lack of long-term follow-up periods
- Heterogeneity of studied CTR interventions with respect to patient selection, duration of the intervention and type of intervention
- The use of surrogate outcome measures (instead of robust outcome measures)

Future CTR interventions should ideally be standardised based on existing CR or CTR guidelines.

First, selection bias has caused CTR trials to mainly include relatively young and male patients with low residual cardiovascular risk (relative to the general population) [22]. Although it is promising that clinical benefits were already demonstrated in these low-risk groups, this implies that the effectivity of CTR has not been thoroughly studied in patients who are traditionally underrepresented in centre-based CR, such as elderly patients, women and patients with comorbidities. The results of the EU-CaRE trial, however, demonstrated that in patients aged 65 years or older who declined participation in centre-based CR, a 6-month CTR programme improved participants’ exercise capacity as compared with patients not participating in CR (between-group difference in VO2_peak_ at 12 months of 0.9 ml/kg/min in favour of CTR) [47]. Moreover, the majority of CTR randomised controlled trials have been conducted in patients with acute coronary syndromes or after coronary revascularisation [21, 22]. The number of trials in patients with CHF has increased in the past decade [23], but the use of CTR in patients with stable angina (as the primary indication for CR) or atrial fibrillation has yet to be investigated. In order to upscale overall CR utilisation, it is highly important that CTR in its studied forms should be applicable to a broad range of patients, including the aforementioned subgroups.

Second, only a few studies have reported on the long-term effectiveness of CTR. Long-term (4 year) follow-up of the FIT@Home trial demonstrated that even in low-risk patients with CAD, their physical fitness and daily PA energy expenditure decreased to levels similar to those before the start of CR [37]. In the Telerehab III trial, however, CTR resulted in a smaller relapse in peak VO2 and subjectively assessed PA as compared with centre-based CR [38], indicating that CTR may prevent part of the relapse in physical fitness and activity that is commonly seen after participation in centre-based CR [35]. Future CTR trials should therefore incorporate prolonged follow-up periods to evaluate this possible advantage of CTR over centre-based CR.

Third, CTR interventions that have been studied are very heterogeneous with respect to patient selection, duration of the intervention and type of intervention [22, 26]. For example, CTR interventions vary greatly with regards to programme content (exercise only or incorporating multiple CR core components), duration (from 6 weeks to 1 year) and technology used to enable data collection (e.g., heart rate monitors, accelerometers, live ECG registration) and remote coaching (e.g., telephone, e‑mail, online applications). This heterogeneity makes it difficult to pool scientific data in order to provide robust conclusions and practical recommendations for the delivery of CTR. We therefore recommend that future CTR interventions be standardised based on CR and CTR guidelines [33].

Finally, most of the published studies used only surrogate endpoints such as peak exercise capacity and PA, but only rarely reported on data about morbidity, rehospitalisation or mortality (or were insufficiently powered to do so). The prognostic impact of the improvement of cardiovascular risk factors and lifestyle behaviour should, however, not be overlooked. A recent study in patients with atherosclerotic cardiovascular disease demonstrated that the improvement of cardiovascular risk factors from current to guideline-directed levels added a median of 7.3 (interquartile range 5.4–10.4) event-free years to a patient’s life [48]. Although additional information about the effectiveness of CTR on more robust endpoints might be needed to persuade policy makers and health professionals about the value of CTR, we believe that—given the current evidence—the implementation of CTR should not be delayed for these reasons.

## Barriers to CTR implementation

Real-life implementations of CTR interventions are relatively scarce, which may be due to several reasons, including 1) uncertainty about ideal candidates for CTR and lack of personalised CTR programmes, 2) lack of reimbursement and concern about investments in CTR, and 3) creation of new workflows and lack of implementation research for CTR.

### Barriers to CTR implementation: **key messages**

- Uncertainty remains about which patients can optimally benefit from CTR interventions
- Lack of reimbursement forms a major barrier to CTR implementation, even though CTR has shown to be cost-effective compared with centre-based CR
- Implementation research is warranted to further investigate implementation barriers and to evaluate the impact of CTR implementation on patients and other stakeholders

Importantly, it remains unclear which patients are optimal candidates for CTR. A recent study demonstrated that several factors were associated with non-participation in CTR for patients with CAD, including higher age, lower educational level and lower exercise capacity and having undergone coronary artery bypass grafting [49]. These results indicated that CTR interventions should be redesigned to better align with the needs and wishes of all patient subgroups. Although digital health should allow for the delivery of such personalised medicine, many CTR interventions still have ‘one-size-fits-all’ approaches, i.e. all patients follow a similar programme, with similar wearable sensors, online applications and methods of coaching, regardless of patient preferences and digital competences. Furthermore, most of the studied interventions focused solely on PA monitoring, while the provision of CTR should ideally contain all CR core components [25]. In addition, CTR programmes should be disease-specific with respect to training volume, educational content and counselling, in order to optimise their effectiveness in underrepresented subgroups such as patients with CHF, atrial fibrillation or stable angina. For instance, exercise training characteristics in patients with CHF should be adapted to lower exercise capacities, and nutritional counselling should involve advice on fluid and salt intake. Essentially, this redesign process should actively involve patients and other stakeholders based on established frameworks for the development of behavioural change interventions [50], ultimately aiming to increase participation and adherences rates, and effectiveness of CTR interventions.

Second, the lack of reimbursement is a major barrier to implementation [51]. In many European countries, reimbursement for CTR interventions is not available, despite their demonstrated cost-effectiveness [3, 22, 52]. This lack of reimbursement may be partly due to the relatively high technological costs documented in most CTR studies, which had rather small sample sizes compared with the overall CR population. One can assume that implementation of CTR will lead to upscaling of technologies applied for its delivery, which will reduce per-capita technological costs and hence improve cost-effectiveness of CTR. Furthermore, it is likely that implementation of CTR results in societal cost savings by increasing overall CR participation. Small studies have indicated that 25–33% of patients not participating in centre-based CR would participate in CTR [23, 24], a percentage that could increase when CTR interventions better meet patients’ preferences. Previous studies have demonstrated that increased overall CR participation and better control of cardiovascular risk factors result in societal cost-savings [4] and a significant amount of added event-free life years [48], which should convince policy makers of the potential return on investment of upscaling overall CR participation rates.

Finally, other important barriers to CTR implementation include the creation of new clinical workflows and lack of implementation research for CTR. Co-creation of novel CTR interventions with all relevant stakeholders, including patients, will improve commitment to new clinical workflows. In this process, it is essential to assess and take into account the digital readiness and digital health literacy levels of end users (both patients and healthcare professionals), as they are important factors in the successful implementation of digital health interventions [27]. To investigate the impact of CTR implementation on patients and other stakeholders, robust implementation research that addresses both the benefits and risks associated with digital health implementation is of eminent importance, but still scarce [53]. Therefore, future CTR research should not only focus on proving and improving the effectiveness of CTR interventions, but also investigate how its implementation affects the quality of care for cardiovascular patients from a broader perspective.

## Conclusions

Cardiac telerehabilitation is a cost-effective alternative to centre-based CR and has the potential to resolve multiple barriers that lead to poor CR completion rates. Moreover, the implementation of CTR programmes could facilitate the extension of traditional CR and secondary prevention programmes in order to improve cardiovascular risk factor management. In order to implement CTR in regular care, CTR interventions need to be standardised and—at the same time—personalised as much as possible, and preferably include multiple core components of CR. Given the current evidence base for CTR, future studies should mainly evaluate the barriers and facilitators for widespread implementation of CTR in regular care.
